# Long-Term Transcriptomic Effects of Prebiotics and Synbiotics Delivered *In Ovo* in Broiler Chickens

**DOI:** 10.1371/journal.pone.0168899

**Published:** 2016-12-21

**Authors:** Anna Slawinska, Arkadiusz Plowiec, Maria Siwek, Marcin Jaroszewski, Marek Bednarczyk

**Affiliations:** 1 Department of Animal Biochemistry and Biotechnology, UTP University of Science and Technology, Mazowiecka 28, Bydgoszcz, Poland; 2 Department of Human Molecular Genetics, Adam Mickiewicz University, Umultowska 89, Poznań, Poland; University of Illinois at Urbana-Champaign, UNITED STATES

## Abstract

*In ovo* delivery of prebiotics and synbiotics in chickens allows for the development of intestinal microflora prior to hatching, which boosts their robustness. The goal of this study was to determine the transcriptomic profile of the spleen (S), cecal tonsils (CT), and large intestine (LI) of adult chickens injected with prebiotics and synbiotics *in ovo*. On day 12 of embryo development, incubating eggs were injected with prebiotics: inulin alone (P1) or in combination with *Lactococcus lactis* subsp. *lactis* IBB2955 (S1), galactooligosaccharides (GOS) alone (P2) or in combination with *Lactococcus lactis* subsp. *cremoris* IBB477 (S2); control group (C) was mock injected with physiological saline. Gene expression analysis was conducted using an Affymetrix Chicken Gene 1.1 ST Array Strip. Most of the differentially expressed genes (DEG) were detected in the cecal tonsils of P2 (378 DEG), and were assigned to gene ontology categories: lymphocyte proliferation, activation and differentiation, and cytokine production. Ingenuity pathway analysis of the DEG (CT of P2) indicated the inhibition of humoral and cellular immune responses, e.g., role of NFAT in regulation of immune responses, phagocytosis, production of nitric oxide, NF-κB, IL-8, and CXCR4 signaling. The DEG with the highest up-regulation from S1 and P2 were involved in gene expression (*PAPOLA*, *RPL27A*, *RPLP1*, and *RPS29*) from P1 and P2 in transport (*BEST4*, *SLC9A3*, and *SLC13A2*), metabolism (*OGT*, *ALPP*, *CA4*, and *CA7*), signaling (*FGG*, *G3BP2*, *UBB*, *G3BP2*, *CACNA1G*, and *ATP6V0A4*), and immune responses (*MSMB*, *LGALS3*, *CABIN1*, *CXCR5*, *PAX5*, and *TNFRSF14*). Two DEG influencing the complement system (*SERPING1* and *MIR1674*) were down-regulated in P2 and S1. In conclusion, GOS injected *in ovo* provided the most potent stimulation of the host transcriptome. This is likely due to its strong bifidogenic effect, which triggers proliferation of indigenous embryonic microflora *in ovo*, and indirectly influences gene expression regulation in host tissues, especially cecal tonsils.

## Introduction

Prebiotics and probiotics contribute to the development of healthy intestinal microflora. Prebiotics are composed of natural, fermentable oligosaccharides that are not digested by the host. On the contrary, they provide a source of metabolizable energy to some genera of the gut bacteria, which indirectly helps to modulate the biodiversity of microflora [1, 2]. Probiotics are defined as microorganisms (typically bacteria) that, once ingested, confer beneficial properties to the host. They control commensal bacteria in the gut, that are recognized by the immune system as “self” [3]. Probiotics contribute to the colonization of intestinal microflora through direct and indirect mechanisms. They produce substrates that stimulate growth of commensals, and antimicrobials to inhibit gut colonization by pathogens, to name a few. They also decrease the permeability of the gut and increase mucin secretion, so that intestinal content does not leak into the system causing chronic inflammation [4]. Prebiotics and probiotics act through quite different mechanisms and in different compartments of the intestine. Therefore they can be combined into one synergistic compound, called a synbiotic [5].

Beneficial properties of prebiotics, probiotics, and synbiotics are manifested by the development of a healthy microbiome, but also by the proper functioning of the immune system, metabolism, and physiology of the host [6]. This intrinsic cross talk between the microbiome and its host is not limited to the gastrointestinal tract (GIT). In fact, the gut communicates with the whole organism by local and systemic immune responses [7] as well as endocrine signals that regulate brain-gut axis [8]. There is considerable evidence of such holistic interactions between microbiomes and the host, based on germ-free [9], dysbiotic [10] or gnotobiotic [11] animal models.

In birds, bioactive compounds can be introduced to a particular compartment of the egg as a solution, using *in ovo* technology [12]. Such an approach can be applied to avian models, which develop in the egg, outside the maternal organism. Thus, avian embryos can be directly manipulated without any developmental damage and/or placental influence and have therefore become a model in biomedical research [13]. For this method to be effective, it is important to select the optimal embryonic stage for the delivery. In chickens, *in ovo* technology has been used mainly on the 18^th^ day of embryonic development (ED) for the intramuscular injection of the vaccines against Marek's disease [14] or on the 17.5^th^ day of ED for *in ovo* feeding of the embryos with the nutrients (e.g., carbohydrates, beta-hydroxy-beta-methyl-butyrate, minerals, vitamins and oligosaccharides) that enhance development of the chicks and growing birds [15–19]. But, for the *in ovo* delivery of prebiotics and synbiotics, an earlier time point, i.e., the 12^th^ day of ED, has been proven empirically, more effective [20]. The injected solution is deposited inside the air cell of the incubating eggs. The prebiotic, due to its high solubility in the water, is transported into the bloodstream and into the developing intestinal tract. The probiotic is, most likely ingested by the embryo during hatching. Birds are hatched with a fully developed microbiome, expressed by the *Bifidobacterium* count in the feces of the chicks [20–23]. So far, we have determined the long-term effects of the *in ovo* delivery of prebiotics and synbiotics on several phenotypic traits, including performance traits, such as body weight, feed intake [21] and meat quality of broiler chickens [24], as well as physiological parameters, including the development of the immune organs [25–27] and pancreatic enzymes [28]. However, the most basic phenotype that reflects the primary reaction of an individual to the particular treatment is the modulation of gene expressions in specific cells or tissues. In our earlier studies, we determined that *in ovo* delivery of synbiotics, consisting of raffinose family oligosaccharides (RFO) and *Lactococcus lactis* subsp., significantly up-regulated *IL-4*, *IL-6*, *IFNB*, and *IL-18* in the spleen (S) and down-regulated *IL-4*, *IL-6*, *IL-8*, *IL-12*, *IFNB*, and *IFNG* in the cecal tonsils (CT) of a native chicken breed at 42 days old [19]. We also detected a down-regulation of immune-related gene expression in the S and CT of broiler chickens injected *in ovo* with inulin or galactooligosaccharides (GOS) combined with *L*. *lactis* subsp., which was more pronounced at the later stages post hatching [29]. These data suggest that the birds respond to the *in ovo* delivery of synbiotics by modulating gene expression levels in immune-related tissues at the later stages post hatching. In this study, we focused on the entire transcriptome responses of the host to prebiotics and synbiotics delivered early during ED. Therefore, we described the long-term molecular adaptations of adult chickens to a single *in ovo* delivery of small doses of prebiotics or synbiotics at the embryonic stage.

## Materials and Methods

### Animals

The experiment was performed on 75 male broiler chickens (Ross 308, Aviangen Inc., Huntsville, AL, USA). Eggs (average weight of 60 g) were obtained from a 32-week-old flock of the broiler breeders. The eggs were incubated for 21 days in commercial settings, using standard conditions (37.8°C and a relative humidity of 61–63%). The eggs were candled on the 12^th^ day of ED to select only viable embryos for *in ovo* injections. On the same day (12 days ED), the eggs underwent *in ovo* delivery of bioactive compounds (prebiotics, synbiotics, or mock injection). Incubation was continued afterwards until hatching. Chicks were sexed, and males (average weight of 42 g) were raised. Separate pens were used for different experimental groups. The animals were raised according to the producer’s rearing instructions. All animals were fed with the same fodder using a three-phase feeding program: starter on days 1 to 14, grower on days 15 to 30, and finisher on days 31 to 34. Feed and water were provided *ad libitum*. The experiment was terminated on day 35. Animal handling methodologies were approved by the Local Ethical Committee for Animal Experimentation, UTP University of Science and Technology in Bydgoszcz, Poland (Permit No 22/2012., June 21^st^ 2012), and were in accordance with the animal welfare recommendations of the European Union (directive 86/609/EEC).

### *In ovo* delivery of prebiotics and synbiotics

Bioactive compounds (prebiotics or synbiotics) delivered *in ovo* were: 1.76 mg/egg inulin prebiotic extracted from *Dahlia tubers* (**P1**) (Sigma-Aldrich GmbH, Schnelldorf, Germany), 0.528 mg/egg GOS prebiotic (trade name: Bi^2^tos) (**P2**) (Clasado Biosciences Ltd., Jersey, UK), synbiotic composed of 1.76 mg/egg inulin and 1000 CFU/egg *Lactococcus lactis* subsp., *lactis* IBB2955 (IBB, PAS, Warsaw, Poland) (**S1**), or a synbiotic composed of 0.528 mg/egg GOS and 1000 CFU/egg *Lactococcus lactis* subsp., *cremoris* IBB477 (IBB, PAS, Warsaw, Poland) (**S2**). The control group (**C**) was mock injected with physiological saline. Doses of prebiotics and synbiotics were optimized *in vitro* using the criterion of bacterial growth in the presence of a given oligosaccharide vs. glucose (data not presented). Immunomodulatory properties of bioactive compounds were assessed with the DT40 cell line stimulated *in vitro* with different prebiotics and bacteria strains [30]. Bioactive compounds were prepared as follows: prebiotics were dissolved in sterile physiological saline, and *L*. *lactis* strains were grown aerobically overnight in a liquid M17 medium at 25–28°C. The concentration of the bacteria was estimated at 3 × 10^8^ living cells per mL. Each synbiotic contained 1000 CFU diluted in physiological saline with prebiotics. A volume of 0.2 mL was used for *in ovo* injections. The shell was punctured with a 0.9 mm needle on the blunt side of the egg. The bioactive solution was deposited inside the air cell using a needle syringe and the hole was sealed with natural glue.

### Sample collection and RNA isolation

On the 35^th^ day, post-hatching, birds from each group (*n* = 5) were randomly selected and euthanized by cervical dislocation. Tissues (S, CT, and large intestine (LI)) were dissected from each bird, snapped frozen in liquid nitrogen, and stored at -80°C for further analysis. Prior to RNA isolation, frozen tissues were homogenized in Trizol (Invitrogen, Carlsbad, USA) using a TissueRuptor homogenizer (Qiagen GmbH, Hilden, Germany). Total RNA was purified using a Universal RNA Purification Kit (EURx, Gdańsk, Poland) according to the manufacturer’s instruction. gDNA residues were removed using a DNase treatment. All RNA samples were evaluated using the NanoDrop 2000 (Thermo Scientific NanoDrop Products, Wilmington, USA) and agarose gel electrophoresis. Additionally, 10% of the samples were analyzed with the Agilent Bioanalyzer 2100 (Agilent, Santa Clara, CA, USA). RNA samples were stored at -20°C before being processed.

### Microarray procedures

Transcriptome analysis was conducted in three tissues (CT, S, and LI) of the chickens in the P1, P2, S1, S2, and C groups (*n* = 5) using Chicken Gene 1.1 ST Array Strips (Affymetrix, Santa Clara, CA, USA). A single-stranded cDNA template was prepared using 100 ng of total RNA with the Ambion WT Expression Kit (Life Technologies, Carlsbad, CA, USA). Fragmentation and labeling of a single-stranded cDNA was conducted with a GeneChip® WT Terminal Labeling Kit (Life Technologies, Carlsbad, CA, USA). The samples were hybridized to the Chicken Gene 1.1 ST Array Strip (Affymetrix, Santa Clara, CA, USA) for 20 h. Next, the array strips were washed and scanned using GeneAtlas® System (Affymetrix, Santa Clara, CA, USA).

### Data analysis

The quality control of the microarray raw data was assessed with Principal Component Analysis (PCA). Whole-transcriptome data were analyzed using Bioconductor [31] implemented in R (version 3.0.2). Pre-processing was done with an oligo package [32]. Microarray data (CEL files) were first loaded into R, and normalized using Robust Multichip Analysis (RMA) algorithm. Normalized data were analyzed using the limma package [33] to identify genes with differential expressions between treatment and control groups. The parameters of the *p*-value ≤ 0.05 and FDR ≤ 0.1 were used to estimate the significance of expressed genes. Cut-off for differentially expressed genes (DEG) was ≥ 1.6 fold change for up-regulated genes and ≤ 0.6 fold change for down-regulated genes. To determine overlapping and non-overlapping DEG between all experimental groups in different tissues, Venn diagrams were generated using Venny [34]. DAVID Gene Functional Classification Tool was used to organize DEG lists into biological modules and assign them Gene Ontology (GO) terms [35, 36]. A threshold of *p*-value < 0.1 was used as a significance level for GO annotations. Lists of GO terms were then batched with CateGOrizer, using two classification methods: GO-Slim and Immune-Related [37]. Pathway analysis of DEG was conducted with an Ingenuity Pathway Analysis (IPA; QIAGEN, Redwood City, CA, USA).

### Validation of microarray with RT-qPCR

To confirm microarray data, 12 DEG were selected for further validation by reverse transcription quantitative PCR (**RT-qPCR**). Total RNA was first reverse transcribed with a Maxima First Strand cDNA Synthesis kit (Thermo Scientific/Fermentas, Vilnius, Lithuania). RT-qPCR reactions were run with a total volume of 10 μL, and consisted of a Maxima™ SYBR Green qPCR Master Mix (Thermo Scientific/Fermentas, Vilnius, Lithuania), 1 μM of each primer, and 280 ng of the cDNA template. Oligonucleotide primers (presented in S1 Table) were designed based on the cDNA sequence to span exon–exon boundaries. NCBI/Primer-BLAST was used to find specific primers [38]. Thermal cycling was done using the LightCycler 480 System (Roche-Diagnostics, Basel, Switzerland) and consisted of initial denaturation at 95°C for 15 min, followed by 40 cycles of amplification: denaturation at 95°C for 15 s, annealing at 58°C for 20 s, and elongation at 72°C for 20s. Fluorescence was measured at the end of each extension step. After amplification, a melting curve was generated by increasing the temperature in small increments up to 98°C, and measuring fluorescence of the melting amplicon. Normalization of the expression levels of the target genes was performed with an ubiquitin C (UB) reference gene. Relative gene expression was calculated with the ^∆∆^Ct algorithm and the amount of the target gene was calculated as 2^–∆∆Ct^ [39]. A student’s t-test was used to determine significance (*p*-value < 0.05).

## Results

### Transcriptome profile

**Fig 1:**
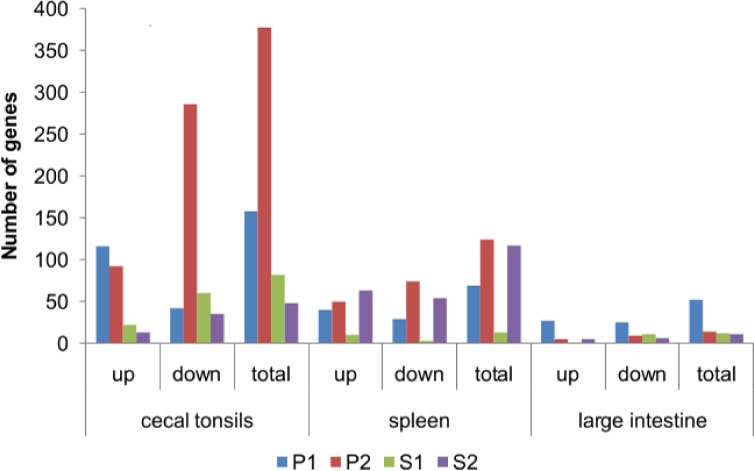
Differentially expressed genes (DEG) in cecal tonsils, spleen, and large intestine of the animals that were treated *in ovo* with prebiotics and synbiotics. Gene expression was determined by whole-genome microarray GeneChip 1.1 ST (Affymetrix, Santa Clara, CA, US) for four substances injected *in ovo*. Experimental groups were injected, *in ovo*, with prebiotics: inulin (P1) and GOS (P2), and synbiotics: inulin with *L*. *lactis* subsp. *lactis* IBB2955 (S1) and GOS with *L*. *lactis* subsp. *cremoris* IBB 477 (S2). The cut-off value was set at 1.66 < FC < 0.66 (FC–fold change). Analysis includes only statistically significant DEG (p-value < 0.05, FDR < 0.1 and). The highest number of DEG was detected in the CT of the P2 group and the lowest in the LI.

A complete set of DEG is reported in S2 Table. A quantitative overview of the DEG number per treatment (P1, P2, S1, and S2) and tissue (CT, S, and LI) is presented in Fig 1. The data were presented as the total number of DEG and broken down to the number of up-regulated and down-regulated genes. In summary, the most DEG were detected in the CT of P2 (378 DEG), with 92 genes up-regulated and 286 genes down-regulated. In the P1 group, 158 DEG were detected in CT, whereas in S1 and S2, 82 and 48 DEG respectively, were detected. In the S, the total number of DEG was lower than in CT and was similar between P2 (124) and S2 (117). P1 had a much lower number of DEG (69), and splenic genes were practically not regulated in S1 (13). In the LI, the total number of DEG was also low. Except for P1 (52), other groups displayed only 11–14 DEG.

**Fig 2:**
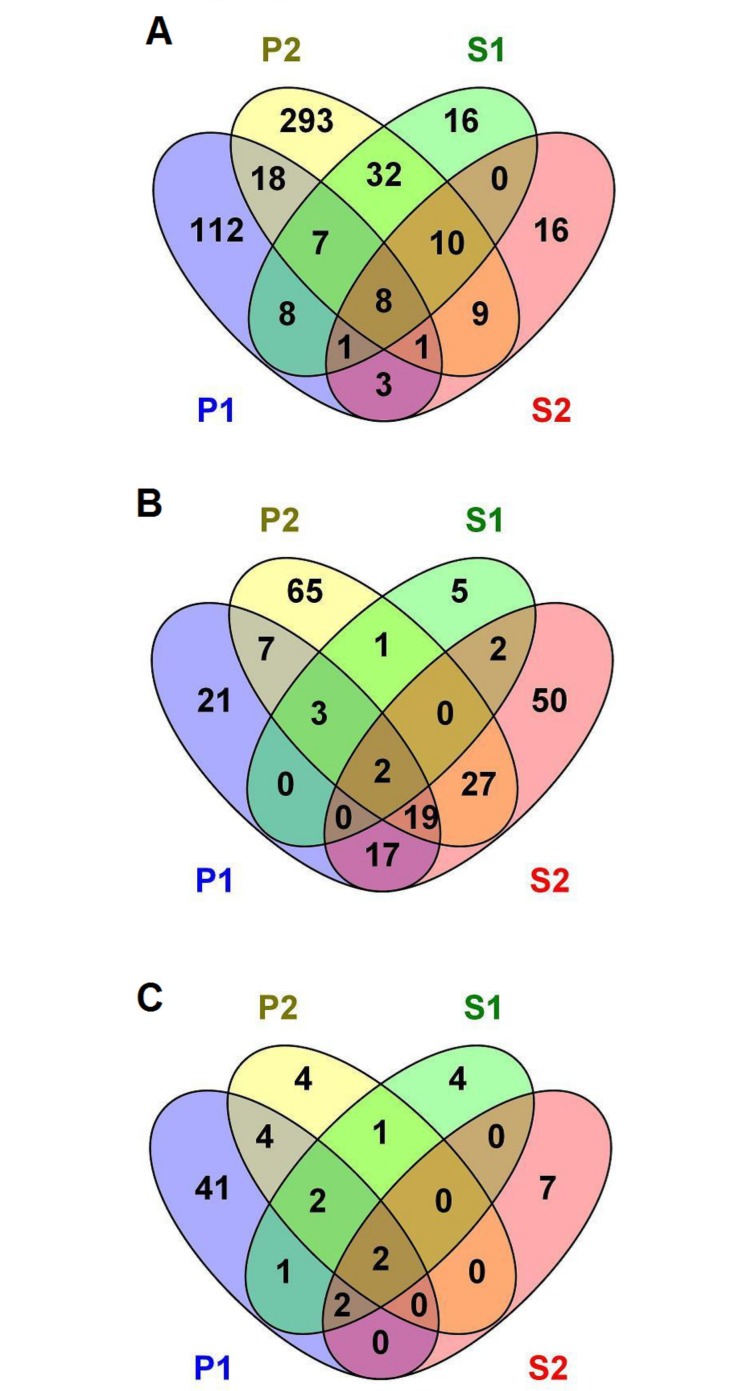
**Venn diagrams: comparison between the number of common and distinct genes between experimental groups in (A) cecal tonsils, (B) spleen, and (C) large intestine.** Experimental groups were injected *in* ovo, with prebiotics: inulin (P1) and GOS (P2), and synbiotics: inulin with *L*. *lactis* subsp. *lactis* IBB 2955 (S1) and GOS with *L*. *lactis* subsp. *cremoris* IBB 477 (S2).

Venn diagrams for DEG are presented in Fig 2. In general, there were more distinct than common DEG across all tissues. In the CT (Fig 2A), the highest number of common DEG was found between P2 and S1 (32 genes), P1 and P2 (18 genes) and P2 and S2 (10 genes). In the S (Fig 2B), the highest number of DEG was shared between P2 and S2 (27 genes), P1 and P2 (19 genes), and P1 and S2 (17 genes). In the LI (Fig 2C), two DEG were shared between P1 and P2, P1 and S2, and P2 and S2. The complete list of common genes is presented in S3 Table.

### Gene ontology

**Fig 3:**
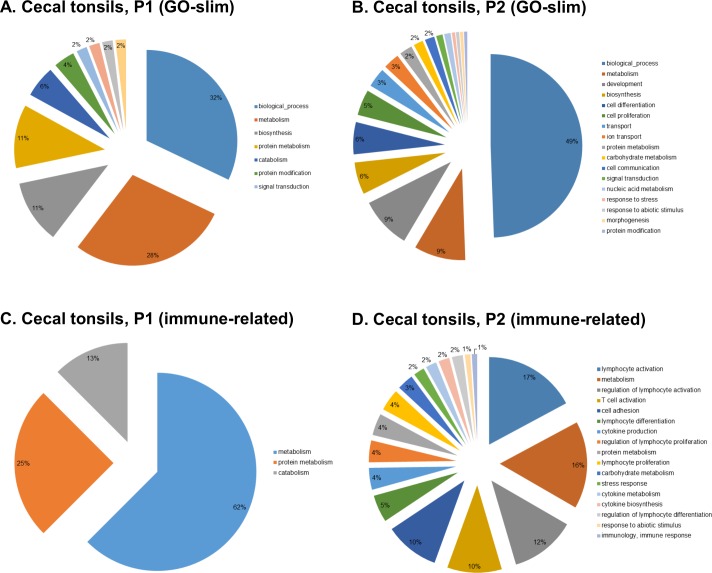
**Categorized GO terms in cecal tonsils of experimental groups injected *in ovo* with prebiotics: inulin (A and B) and GOS (C and D).** GO terms were generated using DAVID (https://david.ncifcrf.gov/home.jsp) and batched into classes with CateGOrizer (http://www.animalgenome.org/cgi-bin/util/gotreei) using GO-slim and immune-related databases.

The complete list of GO terms is presented in S4 Table. Across all experimental groups, 118 GOs were assigned to CT, 47 to the S, and only 6 to the LI. Breaking down the total number of GO into experimental groups, the highest number of GO terms were found in P2 (102), S2 (35) and P1 (34). There were no GO terms assigned to the DEG of the S1 group. To simplify GO term classifications, GO-slim and immune-related databases were used. Only GO identified in the CT of P1 and P2 were categorized successfully and presented in Fig 3. The GO terms from the CT of P1 were batched into a few metabolism-related classes (e.g., metabolism, biosynthesis, catabolism etc.) regardless of the database used. More pronounced categories were identified in the CT of P2. Majority of the GO terms, classified using the GO-slim database, fell within six categories: biological process (49%), metabolism (9%), development (9%), biosynthesis (6%), cell differentiation (6%), and proliferation (5%). Using the immune-related database on GO from CT of P2 allowed for the identification of more specific GO classes, indicating immune system activation. Categories that included most of the GO terms were lymphocyte activation (17%), metabolism (16%), regulation of lymphocyte activation (12%), T cell activation (10%), cell adhesion (10%), lymphocyte differentiation (5%), cytokine production (4%), regulation of lymphocyte proliferation (4%), protein metabolism (4%), and lymphocyte proliferation (4%). The GO term with the largest probability, identified in the CT of S2 (GO:0002376 ~ immune system process), included genes involved in Toll-like receptor signaling pathways (*TLR7* and *TLR15*), TCR signaling (*ORAI1*, *CD4*, *CD28*), BCR signaling (*CTLA4*), NF-ĸB signaling (*IKZF1*), hematopoietic cell lineage (*CD1B* and *CD1C*), TNF signaling (*TNFSF8* and *TNFSF11*), G-coupled protein signaling (*CXCR4*), and cytokine signaling (*SYK* and *IL18*).

### Ingenuity pathway analysis

**Fig 4:**
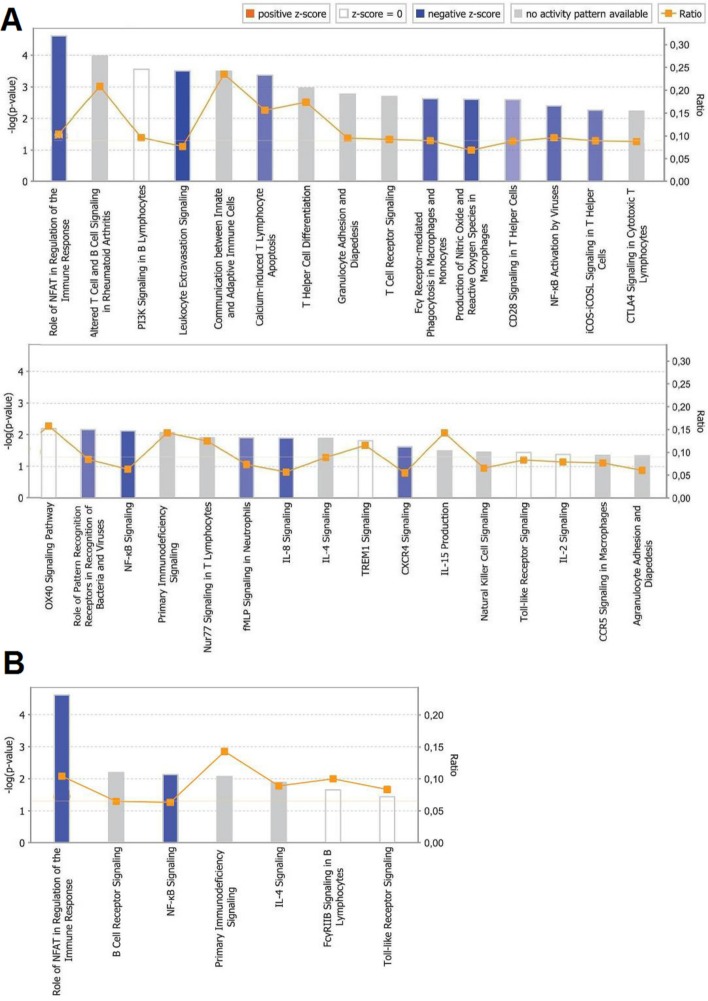
**Ingenuity pathway analysis for (A) cellular immune response and (B) humoral immune response pathways detected in cecal tonsils of chickens injected *in ovo* with GOS prebiotic.** The pathways were identified in IPA software (Qiagen. Hilden, Germany) based on differentially expressed genes (DEG). Upstream regulator analysis was based on the z-score (orange line). Bar color indicates the activation state of the transcription regulators (TR) for the respective pathways: negative z-score (blue bars, TR inhibited), zero z-score (white, no impact on TR) or no activity pattern available (grey bars). No pathways with a positive z-score, which indicates TR activation, were detected. *In ovo* treatment with GOS prebiotic inhibited immune-response pathways in CT.

Ingenuity pathway analysis was conducted for all DEG identified, based on their FC and *p*-values. A sufficient number of DEG were available to identify significant results for the CT of P2. The resulting pathways are presented in Fig 3. In total, 38 canonical pathways associated with immune response, 31 with cellular response (Fig 4A) and seven with humoral response, were identified (Fig 4B). In both cases, a pathway with the highest probability (-log (p-value) > 4) was associated with the role of the nuclear factor of activated T-cells (NFAT) in the regulation of the immune response. Other highly probable pathways (-log(p-value) > 3) in the cellular immune response were altered T cell and B cell signaling in rheumatoid arthritis, PI3K signaling in B lymphocytes, leukocyte extravasation signaling, communication between innate and adaptive immune cells, calcium-induced T lymphocytes apoptosis, and T helper cells differentiation. Remaining entities had lower probability scores (1 < -log (p-value) < 3) in both cellular and humoral immune response pathways.

IPA allows us to calculate the activity pattern of the pathway, expressed by a z-score, which indicates activation (z-score > 0) or inhibition (z-score < 0) of an upstream transcriptional regulator responsible for the modulation of the respective genes. This functionality allowed us to assess that all the pathways detected in the CT of the P2 group were inhibited. For the cellular immune response pathways, a negative z-score was obtained for: the role of NFAT in the regulation of the immune responses, leukocyte extravasation signaling, calcium-induced T lymphocyte apoptosis, Fcγ receptor-mediated phagocytosis in macrophages and monocytes, the production of nitric oxide and reactive oxygen species in macrophages, CD28 signaling in T helper cells, NF-κB activation by viruses, iCOS-iCOSL signaling in T helper cells, the role of pattern recognition receptors in the recognition of bacteria and viruses, NF-κB signaling, fMLP signaling in neutrophils, IL-8 signaling, and CXCR4 signaling (pathways ordered by decreasing probability). For humoral immune response pathways, a negative z-score was detected in the role of NFAT in the regulation of immune responses and NF-κB signaling. The remaining pathways had a z-score equal to zero, or no activity pattern available.

### Genes with the strongest regulation

Tables 1 and 2 present DEG with the strongest regulation in tissues of the chickens injected *in ovo* with prebiotics and synbiotics. Table 1 contains a list of strongly up-regulated DEG (cut-off value: FC > 2.5) and Table 2 contains down- regulated DEG (cut-off value: FC < 0.4). A quantitative comparison between these two tables showed few differences regarding the two datasets; there were 52 strongly up-regulated genes (Table 1) and 26 strongly down -regulated genes (Table 2). The highest number of strongly up-regulated DEG were determined in P1 (30 in total; 18 in CT and 12 in LI) and P2 (12 in total; 6 in CT, 1 in S and 5 in LI). Two genes with the highest FC values across the whole dataset were also detected in the LI of P1: solute carrier family 26 (anion exchanger), member 3 (*SLC26A3*) and solute carrier family 13 (sodium-dependent dicarboxylate transporter), member 2 (*SLC13A2*). On the other hand, the highest number of strongly down-regulated DEG were detected in the P2 (12 genes in total; 9 in CT and 3 in S) and P1 groups (6 genes in total; 1 on CT, 4 in S and 1 in LI). The DEG with the strongest down-regulation were found in S2: myelin-oligodendrocyte glycoprotein-like (*LOC100858813*) CT and myelin-oligodendrocyte glycoprotein-like (*LOC100858813*) LI.

**Table 1:** Differentially expressed genes with the strongest up-regulation.

Gene	Name	ID ^1^	Tissue ^2^	FC ^3^	
**P1. Inulin**					
*MSMB*	microseminoprotein, beta-	423773	**CT**	7.18	
*PAPOLA*	poly(A) polymerase alpha	395878	**CT**	3.57	
*LGALS3*	lectin, galactoside-binding, soluble, 3	373917	**CT**	3.48	
*OGT*	O-linked N-acetylglucosamine (GlcNAc) transferase	422203	**CT**	3.40	
*LOC101750354*	uncharacterized LOC101750354	101750354	**CT**	3.26	
*UBB*	ubiquitin B	396190	**CT**	3.14	
*C9ORF5*	transmembrane protein 245	420957	**CT**	3.08	
*SNORD99*	Small nucleolar RNA SNORD99	ENSGALG00000025389	**CT**	3.02	
*MYO5BL*	myosin-Vb-like	430574	**CT**	2.70	
*RPLP1*	ribosomal protein, large, P1	396262	**CT**	2.65	
*G3BP2*	GTPase activating protein (SH3 domain) binding protein 2	422576	**CT**	2.65	
*ANKS4B*	ankyrin repeat and sterile alpha motif domain containing 4B	427007	**CT**	2.61	
*LOC101752046*	uncharacterized LOC101752046	101752046	**CT**	2.60	
*TUBA1C*	tubulin, alpha 1c	429035	**CT**	2.59	
*NR2F2*	nuclear receptor subfamily 2, group F, member 2	386585	**CT**	2.58	
*C9ORF58*	allograft inflammatory factor 1-like	417179	**CT**	2.55	
*RPL27A*	ribosomal protein L27a	770018	**CT**	2.52	
*RRBP1*	ribosome binding protein 1	396414	**CT**	2.50	
*SLC26A3*	solute carrier family 26 (anion exchanger), member 3	417700	**LI**	15.84	
*SLC13A2*	solute carrier family 13 (sodium-dependent dicarboxylate transporter), member 2	27830	**LI**	14.71	
*CA7*	arbonic anhydrase VII	415791	**LI**	6.66	
*FGG*	fibrinogen gamma chain	395837	**LI**	5.75	
*SLC9A3*	solute carrier family 9, subfamily A (NHE3, cation proton antiporter 3), member 3	20801	**LI**	5.59	
*CACNA1G*	calcium channel, voltage-dependent, T type, alpha 1G subunit	769385	**LI**	4.96	
*BEST4*	bestrophin 4	703847	**LI**	3.41	
*ALPP*	alkaline phosphatase, placental	100859876	**LI**	3.29	
*CA4*	carbonic anhydrase IV	417647	**LI**	3.15	
*SLC22A13L*	solute carrier family 22 member 13-like	420422	**LI**	3.09	
*TMEM252*	transmembrane protein 252	100859235	**LI**	3.01	
*LOC428714*	probable tRNA threonylcarbamoyladenosine biosynthesis protein YwlC-like	428714	**LI**	2.97	
**P2. GOS**					
*MSMB*	microseminoprotein, beta-	423773	**CT**	3.31	
*STEAP1*	six transmembrane epithelial antigen of the prostate 1	420540	**CT**	3.06	
*LGALS3*	lectin, galactoside-binding, soluble, 3	373917	**CT**	2.99	
*SLC26A4*	solute carrier family 26 (anion exchanger), member 4	427845	**CT**	2.93	
*SLC13A2*	solute carrier family 13 (sodium-dependent dicarboxylate transporter), member 2	27830	**CT**	2.88	
*ATP6V0A4*	ATPase, H+ transporting, lysosomal V0 subunit a4	418104	**CT**	2.78	
*C11ORF34*	chromosome 24 open reading frame, human C11orf34	419791	**S**	3.62	
*SLC13A2*	solute carrier family 13 (sodium-dependent dicarboxylate transporter), member 2	427830	**LI**	7.16	
*CA7*	calcium channel, voltage-dependent, T type, alpha 1G subunit	769385	**LI**	6.53	
*CACNA1G*	calcium channel, voltage-dependent, T type, alpha 1G subunit	769385	**LI**	4.05	
*BEST4*	bestrophin 4	703847	**LI**	3.27	
*CA4*	carbonic anhydrase IV	417647	**LI**	2.90	
**S1. Inulin with L. lactis subsp. lactis IBB 2955**					
CUZD1	CUB and zona pellucida-like domains 1	423945	**CT**	2.65	
**S2. GOS with L. lactis subsp. cremoris IBB 477**					
*SNORD20*	small nucleolar RNA, C/D box 20	100498693	**S**	6.45	
*SNORD99*	Small nucleolar RNA SNORD99	ENSGALG00000025389	**S**	3.61	
*CHIR-AB1*	immunoglobulin-like receptor CHIR-AB1	100272165	**S**	2.90	
*RPS29*	ribosomal protein S29	776054	**S**	2.84	
*C9ORF58*	allograft inflammatory factor 1-like	417179	**S**	2.69	
*PCDH15*	protocadherin-related 15	423644	**S**	2.56	
*PAPOLA*	poly(A) polymerase alpha	395878	**S**	2.51	
*GJA1*	gap junction protein, alpha 1	395278	**S**	2.5
*MIR200A*	microRNA 141	777853	**LI**	2.74

^1^ NCBI or ENSEMBL gene ID

^2^ Tissues: cecal tonsils (CT), spleen (S) or large intestine (LI)

^3^ Fold change (FC) > 2.5; p-value < 0.05.

**Table 2:** Differentially expressed genes with the strongest down-regulation.

Gene	Name	ID ^1^	Tissue ^2^	FC ^3^
**P1. Inulin**				
*LOC101748086*	uncharacterized LOC101748086	101748086	**CT**	0.31
*LOC425214*	flagellar attachment zone protein 1-like	425214	**S**	0.20
*LOC100859542*	Uncharacterized protein	Not Available	**S**	0.35
*LOC101748086*	uncharacterized LOC101748086	101748086	**S**	0.38
*SNORA23*	Small nucleolar RNA SNORA23	ENSGALG 00000017862.1	**S**	0.38
*VSNL1*	visinin-like 1	396189	**LI**	0.36
**P2. GOS**				
*LOC425534*	immunoglobulin-like receptor CHIR-B2-like	425534	**CT**	0.24
*CXCR5*	chemokine (C-X-C motif) receptor 5	419784	**CT**	0.35
*SERPING1*	serpin peptidase inhibitor, clade G (C1 inhibitor)	423132	**CT**	0.36
*PAX5*	paired box 5	387330	**CT**	0.36
*SLC6A18*	solute carrier family 6 (neutral amino acid transporter), member 18	100858092	**CT**	0.37
*MRPS5*	mitochondrial ribosomal protein S5	416706	**CT**	0.38
*DRAXIN*	dorsal inhibitory axon guidance protein	419492	**CT**	0.38
*LCN8*	lipocalin 8	396393	**CT**	0.39
*LOC770026*	OX-2 membrane glycoprotein-like	770026	**CT**	0.40
*MRPS5*	mitochondrial ribosomal protein S5	416706	**S**	0.35
*EVI5L*	ecotropic viral integration site 5-like	430998	**S**	0.38
*TNFRSF14*	tumor necrosis factor receptor superfamily, member 14	420403	**S**	0.39
**S1. Inulin with *L*. *lactis* subsp. *lactis* IBB 2955**				
*MIR1674*	microRNA mir-1674	100315777	**CT**	0.31
*MIR1674*	microRNA mir-1674	100315777	**S**	0.36
*LOC769242*	TBC1 domain family member 24-like	769242	**LI**	0.37
**S2. GOS with *L*. *lactis* subsp. *cremoris* IBB 477**				
*LOC100858813*	myelin-oligodendrocyte glycoprotein-like	100858813	**CT**	0.05
*CABIN1*	calcineurin binding protein 1	416938	**CT**	0.34
*LOC425214*	flagellar attachment zone protein 1-like	425214	**S**	0.11
*LOC100858813*	myelin-oligodendrocyte glycoprotein-like	100858813	**LI**	0.04
*LOC769242*	TBC1 domain family member 24-like		**LI**	0.38

^1^ NCBI or ENSEMBL gene ID

^2^ Tissues: cecal tonsils (CT), spleen (S) or large intestine (LI)

^3^ Fold change (FC) < 0.4; *p*-value < 0.05.

### Microarray validation with RT-qPCR

For microarray validation, a set of 12 DEG was selected and analyzed with RT-qPCR. The resulting fold change was compared between the datasets (Table 3). Majority of the genes (10 out of 12) were consistent in their regulation in each treatment. The two genes remaining (*PAPOLA* and *AIF1L*) were regulated in the opposite direction i.e., they were up-regulated in the microarray dataset, but down-regulated in the RT-qPCR dataset.

**Table 3:** Microarray validation with RT-qPCR.

Gene ID	Symbol	Group ^1^	Microarray ^2^		RT-qPCR ^3^	
			FC	*p*-value	FC	*p*-value
**Cecal tonsils**						
100858092	*SLC6A18*	P2	0.37	< 0.001	0.2	<0.05
425802	*CD1B*	P2	0.64	< 0.001	0.42	<0.05
373917	*LGALS3*	P2	2.99	< 0.001	1.47	<0.05
395878	***PAPOLA***	P2	**2.13**	< 0.001	**0.77**	<0.05
427272	*SYK*	S2	0.51	< 0.001	0.6	<0.05
418638	*TLR7*	S2	0.56	< 0.001	0.42	<0.05
420815	*NFATC1*	S2	0.62	< 0.001	0.52	<0.05
417730	*CD36*	S2	1.76	< 0.001	1.62	<0.05
**Spleen**						
417179	***AIF1L***	P2	**2.37**	< 0.001	**0.81**	<0.05
426265	*SFRS7*	P2	2.21	< 0.001	1.49	<0.05
415971	*GP9*	P2	2.23	< 0.001	2.18	<0.05
420899	*SERPINB12*	P2	1.93	< 0.001	1.54	<0.05

^1^ Groups: prebiotic GOS (P2), synbiotic GOS with *L*. *lactis* subsp. *cremoris* IBB 477 FC (S2) (fold change)

^**2**^ Microarray data generated with GeneChip 1.1 ST microarray for GeneAtlas (Affymetrix, Santa Clara, CA, USA) signals were normalized with RMA method

^**3**^ RT-qPCR data generated with custom designed primers used for amplification with SYBR green dye; Ubiquitin C (UB) was used as a reference gene (relative gene expression calculated as 2^–∆∆Ct^). Student's t-tests were used to compare the groups.

## Discussion

In this report, we focused on the microbiome-host interaction in broiler chickens. Specifically, we studied molecular responses of adult broiler chickens to natural promoters of intestinal microbiota (i.e., prebiotics and synbiotics) introduced *in ovo*. Embryonic development in birds takes place inside the egg; this provides an opportunity for the direct manipulation of the embryo and the surrounding membranes. Such manipulation is aimed to promote ED and prepare chicks for post-hatching life (reviewed in [40]). In physiological conditions, avian embryos have limited chances to benefit from contact with the dam during the embryonic and neonatal stages. After being laid, the egg has a definite nutrient composition supporting avian embryo development at all stages (reviewed by [41]). As such, *in ovo* technology is the only method that provides external supplementation to the avian embryo to facilitate its development, and directly increase robustness of the neonate post hatching.

One of the major pitfalls newly hatched chicks face is a poorly developed intestinal health as the gut lacks a microbiome and contains immature gut-associated lymphoid tissue (GALT) (reviewed by [42]). This has serious consequences for post-hatching growth. First, the neonate chick has to switch metabolism from lipids that supported its development during the embryonic stage, to carbohydrates present in the adult-type feed (reviewed by [41]). Secondly, colonization of the neonate gut by a healthy microbiome has to occur as quickly as possible to avoid enteric infections, boost maturation of the immune system, and stimulate better processing of the grain-based feedstuff (reviewed by [43]). In nature, a chick’s microbiome is developed by oral and rectal contact with the dam’s feces and surrounding litter. In commercial hatcheries, sterile environments and no contact with the dam, restrains chicks from having their guts colonized with commensal microbiota. To address the latter issue, we have optimized the *in ovo* delivery of different prebiotics and synbiotics to the chicken embryo on the 12^th^ day ED. On one hand, such an approach promotes healthy microflora in embryonic guts. On the other hand, early inhabiting of the gut by commensal microbiota can stimulate maturation of the cellular and humoral immune responses in central and peripheral immune organs, including GALT [25–27].

In our parallel experiment, a significant influence of the *in ovo* delivery of prebiotics and synbiotics on immune system development was exhibited by an increased proportion of adaptive immune cells within CT [27]. On the 7^th^ day post hatching, CT of all experimental groups (P1, P2, S1, and S2) had been colonized by an increased number of T cells, predominantly helper T cells (CD4^+^) in P2, S1 and S2, and cytotoxic T cells (CD8α^+^) in P1. Increases in the B cells (Bu-1^+^) of 7-day-old chicks was determined in the CT of S1 and S2. At the same time (7^th^ day post hatching), there was a significant depletion of B cells from the bursa of Fabricius in prebiotic and synbiotic-injected chickens (P1, P2, S1 and S2), which suggests the release of the young B cells from primary lymphoid organs and increased colonization of the secondary lymphoid organs (CT) [27]. In 21-day-old chickens, *in ovo* treatments with synbiotics (S1, S2) resulted in a further increase in B cells (Bu-1^+^) in CT. As for S, *in ovo* delivery of synbiotics resulted in increased relative S weight on the 21^st^ day, post hatching [25] and elevated numbers of germinal centers, which are mostly T-independent areas [26]. These findings suggest that synbiotics delivered *in ovo* stimulate the development of mucosal (GALT) and systemic (spleen) humoral immunity in chickens.

### Tissue-specific gene expression

In this study, we analyzed the impact of *in ovo* delivered prebiotics and synbiotics on gene expression modulation in gut and immune-related tissues of adult broiler chickens. The effects of early modulation of chicken embryos depend on the bioactive substance used and the tissue analyzed. As shown above, the treatment that conferred the most pronounced molecular responses on gene expression regulation was the *in ovo* delivery of GOS prebiotics (P2) in CT. The GOS used in our study is a product of enzymatic activity of galactosyltransferases from *Bifidobacterium bifidum* NCIMB 41171 on lactose [44]. This prebiotic has been reported to reduce the invasion or adherence of *Salmonella enterica* serovar *Typhimurium* in murine and porcine *in vitro* and *in vivo* models [44–47].

Avian immune systems lack structured lymph nodes, therefore a major part of the immune responses takes place in S and lymphoid aggregates scattered along the mucosal surfaces i.e., mucosa-associated lymphoid tissue (MALT). The vital part of MALT is located in the gut (i.e., GALT) and comprises of CT, Peyer’s patches, bursa of Fabricius, and numerous lymphoid follicles, present mainly in the hindgut, especially ceca [48]. The CT contains as much as 45.7% of the lymph nodules, and as such, they are the main source of immune function in chicken guts. Furthermore, GALT is linked with the S via common mucosa, which suggests that gut microbiota affect not only local, but also systemic responses [49]. In relation to our data, a vast majority of the molecular responses to *in ovo* treatment was determined in GALT (represented by CT), as opposed to the much lower number of genes activated in S or LI. This can be attributed to anatomical and functional differences between the tissues studied; S is, in general, involved in systemic immune responses, whereas LI is devoid of lymphoid follicles and therefore, its communication with gut microbiota is limited.

### Prebiotics vs. synbiotics

Comparisons between the number of common and specific genes for all groups by Venn diagrams showed that prebiotics (P1, P2) and their respective synbiotics (S1, S2) activated different sets of genes across all tissues. This indicates that prebiotics and synbiotics injected *in ovo* expressed a different mode of action. Synbiotics used for this study were composed based on a three-stage selection procedure, aiming to: (1) determine synergistic interactions between prebiotics and probiotics *in vitro*, (2) assess the immunomodulatory potential of the synbiotic towards host cells *in vitro* [30], and (3) determine doses of prebiotics and synbiotics for *in ovo* injection based on hatchability of chicks and probiotic survival in the chicken GI tract [23].

In spite of this careful selection, differences in gene expression between chickens injected with prebiotics and their respective synbiotics (carrying the same prebiotic component) were quite surprising. GOS prebiotics alone stimulated a more potent gene expression in CT than synbiotics comprised of GOS and *L*. *lactis* subsp. *cremoris* IBB 477. We can explain this phenomenon by the different routes through which prebiotics and probiotics access the embryo after *in ovo* injection. On the 12^th^ day ED, the chorioallantoic membrane (CAM) is strongly vascularized, which enables soluble prebiotics to be transferred gradually via the bloodstream, into the growing embryo. However, probiotic bacteria, because of their size, cannot infiltrate CAM and as such, the chick engulfs them once it is hatched. Since probiotic bacteria that were injected *in ovo* during this study can use respective prebiotics as a source of carbon, we can speculate that they could have metabolized some portion of the prebiotic before it reached CAM. This could explain why the prebiotic alone had a more potent impact on GALT than the same prebiotic in combination with probiotic bacteria.

Even though each treatment activated distinct molecular responses, a few regulated genes were still shared between groups (i.e., 25 DEG shared between P2 and S1 in CT and 19 DEG shared between P2 and S2 in S). Some of those shared DEG reflect the good health of the host. For example, *ATF7* (activating transcription factor 7; down-regulated in P2 and S1 in CT) was first identified in *Caenorhabditis elegans* as the gene involved in the innate immune response to pathogen-specific infections triggered by *Pseudomonas aeruginosa* Exotoxin-A [50]. *MUC13* (Mucin 13 cell surface associated; down-regulated in P2 and S1 in CT) is associated with epithelial barrier function and its overexpression indicates cancer [51]. *FAM26F* (Family with sequence similarity 26, member F; down-regulated in P2 and S1 in CT) has been associated with colitis and is activated during mucosal inflammation [52]. *APOA1 (*Alipoprotein A-I; up-regulated in P2 and S2 in S) is typically down-regulated during Crohn’s disease [53]. Interestingly, *GZMA* (granzyme A), which is a cytotoxic T cell-specific gene identified in the gut epithelium, was up-regulated in P2 and S1 of CT and might represent the activation of cytotoxic T cells (CD8α^+^).

### Gene ontology in the GALT

Analysis of GO terms indicated that when delivered *in ovo*, GOS (but not inulin) had a strong immunomodulatory potential towards gene expression in the GALT. Categorization of the overall GO terms with the immune-related database showed that a majority of the DEG found (CT of P2 group) were involved in lymphocyte activation and differentiation. To elaborate on this, we should consider the phenomenon of the inherent host-microbiome interaction as an integral part of forming avian intestinal health. Chicken GALT is immature at hatching and unable to trigger immune responses [54]. Newly hatched chicks rely mostly on innate immune responses until their gut get colonized with microbiota, which stimulates GALT maturation [55]. There is evidence that during the first few days post hatching, avian GALT undergoes rapid changes [48], including T cell export from the thymus to the periphery and an increase in B cells[27]. Attaining immune functions by GALT in neonate chickens has been associated with the seeding of the GIT with primary microflora, and its evolution to stable climax microflora that assures immune-microbe homeostasis and competitive exclusion of the enteropathogens [56]. The development of the microbial population is strictly age-dependent, which indicates that early stimulation of beneficial microflora is an absolute necessity, as it affects, to a great extent, the entire life-span of an individual [57].

Based on this, *in ovo* delivery of bifidogenic prebiotics (e.g., GOS) into embryonic GALT is an efficient way to promote the proliferation of the indigenous microflora and faster maturation of the intestinal immune responses. In this study, we provide evidence that *in ovo* inoculation with prebiotics contribute to life-long GALT functions, expressed by a long-term regulation of immune-related pathways. GO terms identified in the CT of the P2 group indicates that the adult broiler chickens have developed adaptive immune responses. In immunologically mature GALT, the most efficient strategy to protect the gut from enteric bacteria is the neutralization of antigens from being bound by epithelial cells [58]. This is achieved by secreting neutralizing antibodies by intestinal epithelial lymphocytes (IEL) into the intestinal lumen. Gene expression regulation identified in the GALT of P2 chickens suggests that the processes involved in adaptive immune responses (cellular and humoral) are regulated at the molecular level.

### Negative regulation of the immune-related pathways

Based on pathway analysis, we infer that the transcriptional regulation of immune-related genes in the CT of P2 is oriented towards the down-regulation of cellular and humoral immune pathways in GALT of adult broiler chickens. One of the main functions of GALT is to develop a tolerance towards beneficial microflora along with the potential for fighting enteric pathogens [59]. The major benefit of such an evolutionary adaptation is that the healthy microbiome, which is tolerated by the host, prevents pathogen colonization in the GIT [60]. At the same time, the host does not need to allocate energy into mounting inflammatory and acute phase immune responses, which is not only energy demanding, but can also have harmful side effects. However, it is noteworthy that such down-regulation of immune-related genes, in response to *in ovo* injections of prebiotics or synbiotics, does not happen instantly at hatching. In our previous studies we determined that cytokine gene expression decreases as chickens age and is most apparent in the oldest animals (in this case, 5-weeks-old broiler males) [29]. It led us to a conclusion that the maturation of the microbiome is a prerequisite for the exertion of observable effects on the host.

### Genes with the strongest regulation

#### Up-regulated genes–housekeeping, signaling & immune pathways

DEG with the highest mRNA abundance were defined in the P1 and P2 groups and are involved in basic housekeeping processes, such as gene expression, transport, metabolism, as well as signaling and immune responses (gene function based on http://www.genecards.org). Regulators of gene expression include Poly(A) polymerase alpha (*PAPOLA*), which is responsible for adding Poly(A) at the 3'end of pre-mRNA; members of the CFTR translational fidelity (class I mutations) pathway (e.g., *RPL27A*, *RPLP1* and *RPS29*), which prevent the recoding of nucleic acids during translation and modification of the amino acid sequence; and small nuclear RNA genes (e.g., *SNORD20* and *SNORD99*), which can modify sequences of substrate RNA through methylation. These genes maintain protein synthesis and stimulate ribosomal proofreading.

DEG involved in metabolic processes were strongly up-regulated in the LI of P1 and P2. They include O-linked N-acetylglucosamine (GlcNAc) transferase (*OGT*), alkaline phosphatase, placental (*ALPP*) and carbonic anhydrase IV and VII (*CA4* and *CA7*). The latter is of particular interest because it has been reported in ruminants as a marker for a healthy follicular-associated epithelium (FAE) [61]. FAE is known to absorb pathogens and transport them to underlying lymphatic tissue [62]. These cells can actively secrete carbonic anhydrase, which is an enzyme responsible for the reversible conversion of carbon dioxide into bicarbonate. This way it maintains the acid-base balance in the blood. Furthermore, it is a marker of intestinal health, since an FAE that absorbs pathogens undergoes lesions and stops producing the enzyme.

Another group of up-regulated DEG (e.g., *BEST4*, *SLC9A3* and *SLC13A2*) takes part in pathways involved in the transport of glucose and other sugars, bile salts, organic acids, and amine compounds. These genes encode for transporter proteins that allow sugars and other metabolites to enter and exit cells. Increased expression of sodium-dependent glucose co-transporters (SGLTs) in the intestinal tissues (LI and CT) of chickens injected *in ovo* with prebiotics (P1 and P2) indicates their enhanced ability to absorb monosaccharides. It also corresponds with a high increase in the pancreatic potential for production of amylase that was identified in the same animals at 21 days old [28].

Finally, up-regulated DEG were also involved in signaling and immune pathways such as integrin signaling (*FGG*), TNF-alpha signaling (*G3BP2*), NF-ĸB signaling (*UBB* and *G3BP2*), T-cell receptor signaling (*CACNA1G*), and epithelial signaling in *Helicobacter pylori* infection (*ATP6V0A4*) pathways. Members of immune-related DEG, beta inhibin (*MSMB*) and lectin galactoside-binding soluble 3 (*LGALS3*), were highly up-regulated in the CT of prebiotics-treated groups (P1 and P2). Both genes (*MSMB* and *LGALS3*) are important regulators of immune processes. Inhibin members of the *TGF-β* superfamily regulate T cell selection and stromal cell differentiation [63], whereas *LGALS3* plays a role in innate immunity, including the gastric infection of *Helicobacter pylori* [64].

#### Down-regulated genes–immune signaling & complement

Among strongly down-regulated DEG (in CT of P2 and S2 groups), there are genes involved in G-coupled protein receptor signaling (*CABIN1* and *CXCR5*), nuclear factor of activated T cell (NFAT) pathways (*CABIN1*), chemokine signaling (*CXCR5*), lymphocyte signaling (*PAX5*), and tumor necrosis factor signaling (*TNFRSF14*) pathways (gene function based on http://www.genecards.org). Interestingly, two DEG, which are responsible for the regulation of the complement pathway, were inhibited in the CT (P2 and S1) and S (S1). The complement system is an active part of an innate immunity and a connection between innate and adaptive immune responses. First, *SERPING1*, a gene that was strongly down-regulated in the CT of P2, encodes for a protein that is a C1 inhibitor and negatively regulates the C1 component of the complement cascade (lectin cascade). Secondly, the S1 group had a down-regulated expression of *MIR1674*, a miRNA that has 54 predicted targets (based on www.mirdb.org). One of the highly probable targets of *MIR1674* is the CUB and Sushi multiple domains 1 (*CSMD1*) gene, which is also a complement inhibitor. In this case, however, down-regulation of *MIR1674* was followed by *CSMD1* activation and, consequently, complements inhibition. Thus, we can conclude that the complement pathway regulation may be involved in host-microbiome interactions.

#### Microarray validation with RT-qPCR

Microarray results corresponded with the RT-qPCR used for validation; 10 out of 12 DEG had confirmed expression. Two of the genes that were expressed differently in two methods were poly (A) polymerase alpha (*PAPOLA*) and allograft inflammatory factor 1-like (*AIF1L*). In the microarray, they were up-regulated and in RT-qPCR down-regulated. *PAPOLA* is a large gene, with 22 coding exons and a transcript length of 3’084 bps. *AIF1L* has five coding exons, but a very similar transcript length, 2’523 bps. Based on the records in the ENSEMBL database (http://www.ensembl.org), both genes have only one transcript variant, therefore, this discrepancy in gene expression estimation does not result from alternative splicing. On one hand, results of RT-qPCR are usually considered more accurate for estimating gene expression, since microarray hybridization results can be biased for gene families or genes that have similar fragment sequences connecting the probe on the microarray. On the other hand, both techniques vary in coverage with oligonucleotide primers/probes. In the microarray, each transcript hybridizes 26 probes on average, whereas in RT-qPCR, only one primer pair is used per transcript, spanning over two exons. This is why some inconsistencies will always appear between these techniques. For higher accuracy, we should not focus on single genes, but rather on the entire groups of genes or pathways, because they will have more of a statistical and biological impact.

## Conclusions

In this paper, we have analyzed long-term transcriptomic responses in immune and gut tissues of chickens that were injected *in ovo* with prebiotics and synbiotics. Of four bioactive compounds delivered *in ovo*, GOS proved to be the most potent one in the stimulation of the host–microbiome interactions. The strong bifidogenic effect of GOS triggered a strong down-regulation of immune-related genes and pathways in CT. The observed phenomenon was possible because, GOS prebiotics delivered *in ovo* on day 12 ED was able to infiltrate CAM in the egg and stimulate the growth of indigenous microbiota in the embryonic GIT. These microbiota boosted the maturation of GALT, which resulted in the enhanced tolerance of the local immune system. The transcriptomic effects were expressed in the inhibition of cellular and humoral immune responses. This indicates that the regulation of the complement pathway could be one of the proposed mechanisms of negative regulation of the inflammatory immune responses in adult birds that received the GOS prebiotic *in ovo*.

## Supporting Information

S1 Table Primer sequences for microarray validation with RT-qPCR (XLSX)

S2 Table Differentially expressed genes (DEG) in cecal tonsils, spleen, and large intestine of adult chickens injected with prebiotics and synbiotics *in ovo*. (XLSX)

S3 Table Common and specific genes between different experimental groups of chickens injected with prebiotics and synbiotics *in ovo*. (XLSX)

S4 Table Gene ontology (GO) terms associated with the genes regulated in adult chickens that were injected with prebiotics and synbiotics *in ovo*. (XLSX)
